# Water as a Sanitary Agent

**Published:** 1864-02

**Authors:** 


Water as a Sanitary Agent.—General Morin, who has much occupied himself with improvements in the ventilation of public buildings, in a note addressed to the Academy of Sciences, in Paris, treats of what he terms the "hygrometricity" of confined places. Much struck with the importance which the English engineers and authors attach to the imparting to the air employed for ventilation, whether heated or not, a certain amount of moisture, he was induced to investigate whether the salubrity of such air might not be due in some measure to the development of a certain amount of electricity by the passage of the air through the vaporized water (as in the case with regard to dew and rain during storms), giving rise to the production of free oxygen. If this or some analagous modification can be shown to take place, we have in our hands a simple, efficacious, and economical means of purifying the air of inhabited places, especially in summer. Air containing free oxygen, possesses in a high degree the property of burning certain miasmata and emanations from bodies in a state of putrefaction. General Morin accordingly instituted some experiments, in order to ascertain whether the dispersion or solution of a certain quantity of sprayed water in the air sensibly modified its electrical condition. The results show the extrication of free oxygen, and the subsequent or concurrent formation of an acid. As both the oxygen and the acid, probably a nitrous compound, possess the property of destroying putrefactive emanations, their presence sufficiently proves that the vaporization of water in the air, besides the increase of moisture and depression of temperature which it gives rise to, may exert an action on the animal economy, and upon the air of habitations, deserving the attention of sanitarians.—*Sanitary Reporter.*

---

The influence of the cotton famine on the rate of mortality was the subject of a lecture by Dr. Noble at the last annual meeting of the Statistical Society of Manchester. He said he had the best grounds for stating that for the last two years there had been no unusual mortality in the cotton districts. From the last census it appears that there are, in England and Wales, one surgeon or general practitioner to about 1,712 of the population, one physician to 5,532, and one dentist to 3,505.—*London Lancet.*

